# Synthetic signal sequences that enable efficient secretory protein production in the yeast *Kluyveromyces marxianus*

**DOI:** 10.1186/s12934-015-0203-y

**Published:** 2015-02-14

**Authors:** Tohru Yarimizu, Mikiko Nakamura, Hisashi Hoshida, Rinji Akada

**Affiliations:** Department of Applied Molecular Bioscience, Yamaguchi University Graduate School of Medicine, 2-16-1 Tokiwadai, Ube, 755-8611 Japan; Innovation Center, Yamaguchi University, 2-16-1 Tokiwadai, Ube, 755-8611 Japan; Present address: Environmental Biofunction Division, National Institute for Agro-Environmental Sciences, 3-1-3 Kan-nondai, Tsukuba, Ibaraki 305-8604 Japan

**Keywords:** Synthetic signal sequence, Hydrophobic core, Secretion, Thermotolerant yeast, Heterologous protein production

## Abstract

**Background:**

Targeting of cellular proteins to the extracellular environment is directed by a secretory signal sequence located at the N-terminus of a secretory protein. These signal sequences usually contain an N-terminal basic amino acid followed by a stretch containing hydrophobic residues, although no consensus signal sequence has been identified. In this study, simple modeling of signal sequences was attempted using *Gaussia princeps* secretory luciferase (GLuc) in the yeast *Kluyveromyces marxianus*, which allowed comprehensive recombinant gene construction to substitute synthetic signal sequences.

**Results:**

Mutational analysis of the GLuc signal sequence revealed that the GLuc hydrophobic peptide length was lower limit for effective secretion and that the N-terminal basic residue was indispensable. Deletion of the 16th Glu caused enhanced levels of secreted protein, suggesting that this hydrophilic residue defined the boundary of a hydrophobic peptide stretch. Consequently, we redesigned this domain as a repeat of a single hydrophobic amino acid between the N-terminal Lys and C-terminal Glu. Stretches consisting of Phe, Leu, Ile, or Met were effective for secretion but the number of residues affected secretory activity. A stretch containing sixteen consecutive methionine residues (M^16^) showed the highest activity; the M^16^ sequence was therefore utilized for the secretory production of human leukemia inhibitory factor protein in yeast, resulting in enhanced secreted protein yield.

**Conclusions:**

We present a new concept for the provision of secretory signal sequence ability in the yeast *K. marxianus*, determined by the number of residues of a single hydrophobic residue located between N-terminal basic and C-terminal acidic amino acid boundaries.

**Electronic supplementary material:**

The online version of this article (doi:10.1186/s12934-015-0203-y) contains supplementary material, which is available to authorized users.

## Background

The signal sequence for a secretory protein is the first designated peptide sequence that exhibits similarity with the common amino-acid domain located at the N-terminus of all secretory proteins [1-3]. The signal peptide usually consists of an N-terminal basic residue and a subsequent stretch of amino acids containing a hydrophobic core, known to be recognized by the signal recognition particle in both prokaryotes and eukaryotes. In eukaryotes, the signal recognition particle translocates the proteins to the inner side of the endoplasmic reticulum (ER) [1,4-8]. After insertion into the ER, the proteins are trafficked to the Golgi body and secretory vesicles; finally, membrane fusion of the secretory vesicles to the plasma membrane excretes the proteins into the extracellular environment. Whereas the N-terminal signal sequence is indispensable for protein secretion, strict consensus sequence has not been found. Generally hydrophobic amino acids in the region number from ten to fifteen, but not fewer than six.

The hydrophobic amino acids are Leu, Ile, Val, Phe, Ala, Trp, Met, and Gly, and these exhibit similar chemical characteristics. Consequently, by calculating the hydrophobicity values for each amino acid, probable signal sequences can be predicted by software programs [9-12]. However, it is also known that different signal sequences exhibit different levels of secretory activity [13-16]. Studies have found that replacement of the original signal sequences of foreign proteins with that of the host organisms has resulted in an enhancement of heterologous protein production [13,15,17-19]. For example, the addition of the N-terminal region of the α mating factor to heterologous proteins enhanced secretory protein production in yeast [17,18,20,21]. These results indicate that there may be a preference for signal sequences among different organisms. In order to develop a strategy for efficient secretory protein production for industrial purposes, and also to reveal a potential mechanism underlying the role of signal sequences in protein secretion, we attempted to model secretory signal using simplified, synthetic peptide sequences.

For the purpose of creating numerous synthetic peptide sequences by recombinant DNA technology, a recently developed non-homologous end joining (NHEJ) cloning system was applied in this study. The yeast *Kluyveromyces marxianus* exhibits efficient NHEJ activity that joins DNA ends in a sequence-independent manner through transformation [22]. When DNA fragments are prepared by using primers with synthetic sequences to attach to the N-terminus of a protein, these fragments can be autonomously circularized by NHEJ following introduction into the yeast. Therefore, DNA constructs containing synthetic signal peptide sequences can be created and expressed for examination of their secretory activities only through transformation of the PCR products into *K. marxianus*.

By comprehensive mutational analysis of the N-terminal sequence of the GLuc luciferase gene, we found that the presence of the acidic Glu residue downstream of the hydrophobic peptide had a role in defining the boundary of the signal sequence. Through placement of N-terminal basic and C-terminal acidic amino acids on either side of a hydrophobic core, we were able to examine various synthetic amino acid stretches for their secretory protein production in yeast. The results indicated that the number of amino acids adequate for efficient secretion could be defined when a single hydrophobic amino acid repeat was utilized instead. Unexpectedly, we found that the sixteen of the same amino acid (Met) provided the most efficient secretory production of GLuc in the yeast *K. marxianus*.

## Results

### Deletion analysis of the N-terminal sequence of yGLuc

**Figure 1:**
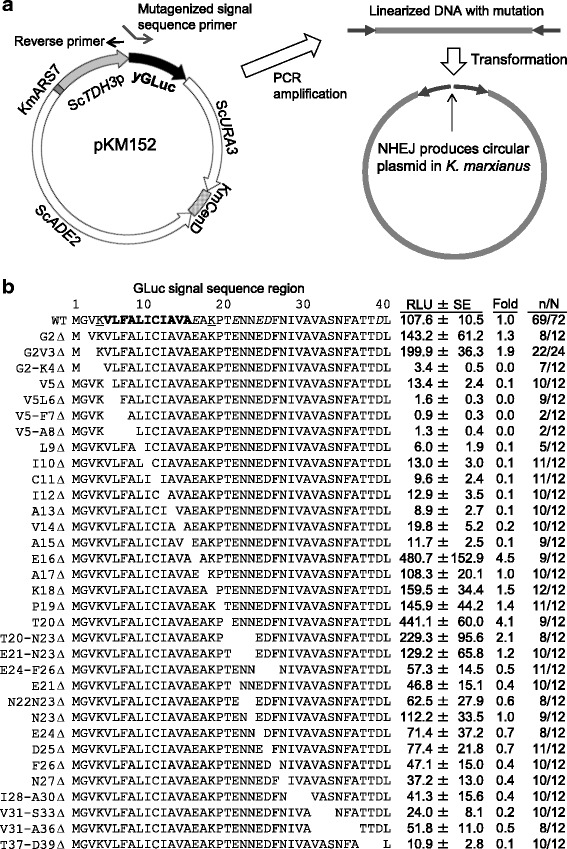
**Deletion analysis of the GLuc signal sequence. a)** The pKM152 plasmid structure is shown. Non-homologous end joining in *K. marxianus* produces circular plasmid efficiently, that makes deletion and mutagenized sequence addition experiments easier and faster. **b)** Deletion series of GLuc signal sequences and associated levels of secretory GLuc activity. The N-terminal signal sequence of GLuc has a hydrophobic amino acid stretch (bold letters in wild-type sequence (WT)) between the 4th Lys (K) and the 16th Glu (E). Positively and negatively charged amino acids are underlined and italicized, respectively. RLU; relative luminescence units (value/(μl · sec · OD_600_)), Fold; ratio of mutagenized GLuc activity per wild type activity. n; number of transformants used for calculation, N; number of transformants measured.

To determine the amino acids essential for the secretory production of yGLuc, we performed deletion analysis of the signal sequence (Figure 1). The N-terminal 17 amino acid sequence was indicated as the signal sequence of GLuc (New England Bio Labs, Inc.)

Luciferase activities generated using the deleted signal sequences are shown in Figure 1b. Deletion of the 2nd Gly (G) and 3rd Val (V) did not cause significant changes but the additional deletion of the 4th Lys (K) diminished the activity level, indicating that this K residue is important for secretion activity. In addition, deletions of the following amino acids individually or in multiples in the sequence VLFALICIAVA (Leu: L; Phe: F; Ala: A; Ile: I; Cys: C) also diminished the activity, indicating the importance of the hydrophobic core sequence. Contrary to these results, the deletion of the 16th Glu (E) enhanced the activity considerably. Deletion of the 17th A showed an activity level comparable with that of the wild type, and deletion of the 18th K also showed increased activity. These results suggested that the 16th E and 18th K inhibited secretory activity in *K. marxianus*. Deletion of the amino acids downstream from the 18th K decreased the activity slightly in a gradual manner except for 20th T, which increased the activity.

### Substitution of the 16th E and the 4th K of the yGLuc signal sequence

**Figure 2:**
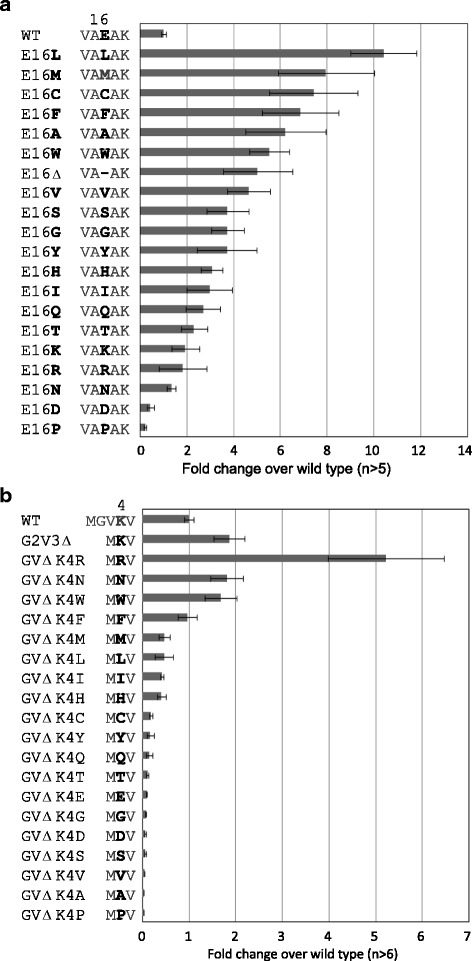
**Effect of amino acid substitution in the GLuc signal sequence on secretion activity. a)** Mutagenesis of the 16th Glu (E). E16Δ is the deletion mutant and the others are substitutions to other amino acids. **b)** Mutagenesis of the 4th Lys (K), with deletion of the 2nd Gly and 3rd Val (GVΔ). Activity is shown as fold change calculated as the ratio of mutagenized GLuc activity per wild type activity. The error bars indicate SEs of at least 5 **(a)** or 6 **(b)** transformants except for null-activity ones.

To define a role for the 16th E of the signal sequence, we substituted the 16th E with each of the other amino acids (Figure 2a). Substitution to L increased the activity more than ten-fold. Similarly, substitution to Met (M), C, F, A, Trp (W), or V increased the activity to more than four-fold than that of wild type. In contrast, substitution of E to Asp (D) or Pro (P) decreased the activity, indicating that these amino acids may have a similar (inhibitory) role to E, which may define a boundary of hydrophobic core.

Similarly, the 4th K was substituted to each of the other amino acids (Figure 2b). In this case, the 2nd G and the 3rd V were deleted as well. Substitution of K to Arg (R) increased the measured luciferase activity, and Asn (N) and W substitutions yielded levels similar to the original K. However, all other amino acids showed decreased activities upon substitution, suggesting that the presence of specific amino acids at the N-terminus of hydrophobic core is necessary for secretion. From these results, we assumed that the hydrophobic core was defined by the region between the N-terminal basic and C-terminal non-hydrophobic amino acids.

### Substitution of the hydrophobic core with a single amino acid stretch

**Figure 3:**
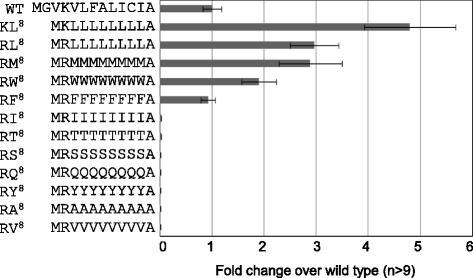
**Substitution of the hydrophobic stretch to eight repeats of single amino acids.** Poly-Leu (L), Met (M), Trp (W), and Phe (F) increased the secretory luciferase activities, but other substitutions showed no activity levels. Data were shown as in Figure 2.

The hydrophobic core from the 5th to 15th residues in the yGLuc N-terminus was found to be necessary for secretion, and this region was defined by the presence of the 4th K and the 16th E. Deletions of only a single amino acid, such as V5Δ, L9Δ, I10Δ, C11Δ, I12Δ, A13Δ, V14Δ, and A15Δ in Figure 1b, were sufficient to damage the total secretion of GLuc, suggesting that the hydrophobic core of GLuc signal sequence may be the minimum required for secretion in yeast, when defined by the 4th K and the 16th E. Next, we attempted to substitute a part of the hydrophobic core with a stretch of single repeated amino acids. Since the 2nd G and 3rd V were not necessary for the activity, these were deleted. The eight amino acid sequence of VLFALICI from the 5th through 12th sites was replaced with eight consecutive single repeated amino acids (Figure 3); for example, a LLLLLLLL stretch is expressed as L^8^. The KL^8^ and RL^8^ substitutions showed higher activities than wild type; thus, R was placed before a stretch for a subsequent experiment. The results of this showed that RL^8^, RM^8^, RW^8^, and RF^8^ exhibited elevated activities, but R followed by stretches of I, Thr (T), Ser (S), Gln (Q), Tyr (Y), A, or V showed very low activities (Figure 3). We constructed and examined the RC^8^ construct as well, but almost all transformants showed the level of null value (data not shown), and thus were not included in the result. The substituted stretches constituted of L, M, W, or F may have led to stronger activity than the VLFALICI hydrophobic core but the activity of other stretches, such as I, T, S, Q, Y, A, and V, was weaker than that of the core. The results also indicated that single amino acid stretch can be used as a signal sequence.

### Hydrophobic core designation using various lengths of a single amino acid

**Figure 4:**
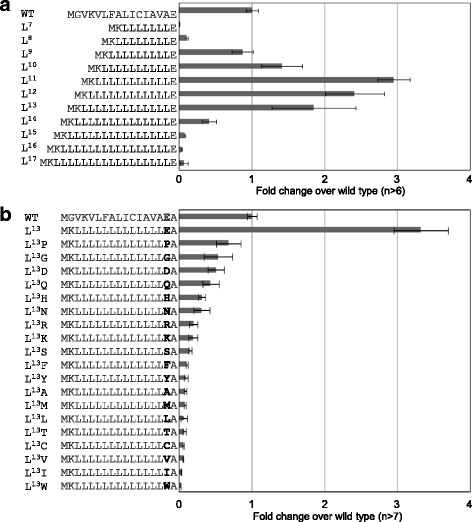
**Effect of L repeat number and of the terminal amino acid following poly-L** ^**13**^ **. a)** The length of the poly-L sequence between the N-terminal K and C-terminal E was changed from seven to seventeen. **b)** The E following the poly-L^13^ was substituted to other amino acids. Data were shown as in Figure 2.

As the hydrophobic core can be replaced effectively by a stretch of Ls (Figure 3), the VLFALICIAVA sequence located between the N-terminal K and the C-terminal E was substituted with stretches consisting of various lengths of L (Figure 4a). L^7^ and L^8^ did not show secretory activity but from L^9^ and longer, the activities were increased. The best activity was observed in L^11^. From L^13^ or longer, the activities decreased remarkably. This result suggested that there is suitable hydrophobicity for efficient secretory production determined by a specific number of amino acids.

To determine effective amino acids as the boundary for the hydrophobic core, Glu following to poly-L was substituted with the other amino acids. We used L^13^ instead of L^11^ for the border-E substitution experiment because loss of the boundary will show lower GLuc activity by hydrophobic sequence extension (Figure 4b). Substitution for E with any other amino acid decreased secretory activity, indicating that E is the most effective amino acid at the boundary of hydrophobic core. The placement of E at the C terminus of the hydrophobic core may have a strong role in determining the extent of the hydrophobic region. Overall, we speculate that an effective secretion signal peptide requires an adequate hydrophobic core, which is experimentally determined by the flanking N-terminal basic and C-terminal E residues. Stronger or weaker hydrophobicity in this region may be inadequate for efficient secretion.

### Optimal amino acid number for a hydrophobic core

**Figure 5:**
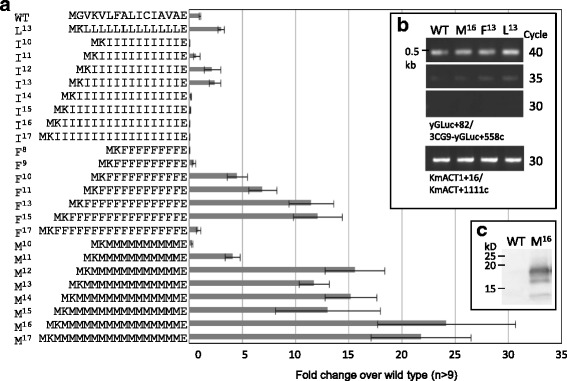
**Effect of repeat number of the single amino acid between K and E on secretory activities. a)** The poly-I, F, and M sequence was changed to 10–17, 8–17, and 10–17, respectively and the activities were compared with WT signal sequence. Data were shown as in Figure 2. **b)** RT-PCR of the sequenced clones for WT (RAK6205), M^16^ (RAK8772), F^13^ (RAK9383) and L^13^ (RAK10336). Amplified cDNA bands of yGLuc and the control *ACT1* at 30, 35, 40 cycles are shown. **c)** Western blotting analysis of the supernatants from WT (RAK6205) and M^16^ (RAK8772) using anti-GLuc antibody.

The previous results suggested that efficient secretion might be defined by a specific number of single amino acids between the N-terminal basic K and C-terminal E amino acids in the signal sequence. Therefore, we placed various lengths of poly-I, -F, or -M between the N-terminal K and C-terminal E in the signal sequence (Figure 5a). As shown in Figure 3, substitution to RI^8^ did not show any activity. However, I^12^ and I^13^ showed elevated activity similar to that of L^13^ (Figure 5a). In the case of poly-L, nine to thirteen repeats showed high activity, but for poly-I, only twelve and thirteen showed activity.

Similarly, we varied the amino acid length with poly-F and poly-M. For these constructs, the trend was similar to that seen with poly-L, but the activity was increased substantially. The F^13^ and F^15^ substitutions showed a eleven-fold increase over the wild type, and M^16^ showed a twenty-four-fold increase. Interestingly, poly-M showed these extremely elevated activity levels over a wide range; from twelve to seventeen Ms, all showed more than twelve-fold enhancement over the original sequence, though native poly-M sequences are rarely found in the protein database (see the Discussion section).

All of the constructs expressed the modified yGLuc under the control of the Sc*TDH3* promoter. However, it is possible that the differences in the activity levels were caused by differences in the levels of transcription. To examine the levels of transcripts produced by the constructs (Figures 4a and 5a), total RNA was isolated from these strains and RT-PCR was performed with 30, 35, and 40 cycles using the primers for yGLuc and *ACT1* as a control (Figure 5b). All showed similar band intensities, indicating that mRNA levels were roughly similar in these strains.

The culture supernatants of the wild type and M^16^ strains were examined by western blotting using anti-GLuc antibody (Figure 5c). Only M^16^ supernatant showed an intensive band at a smaller size than 20 kD. The predicted molecular weights of M16:GLuc were 20.7 kD with M^16^ signal sequence and 18.4 kD without the signal sequence. The detected protein size of the western blotting analysis suggests that the M^16^ signal sequence may be cleaved. The activities in culture supernatant and culture fluid containing yeast cells were comparable (data not shown), indicating that the GLuc consisting of M^16^ signal sequence were actually released from cells.

### Heterologous signal sequences

**Figure 6:**
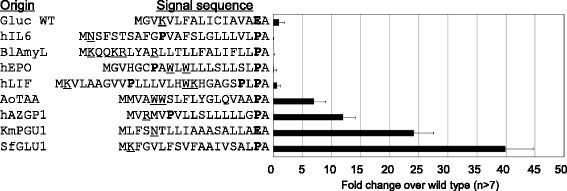
**Substitution of the GLuc signal sequence with heterologous and homologous signal sequences.** The GLuc signal sequence, up to the 16th E, was substituted with other signal sequences by connecting the C-terminal P of the other signal sequence to the site of the 16th E of GLuc. N-terminal K, R, N, and W are underlined and P and E are shown in bold letters. Signal sequences were utilized from hIL6: human interleukin 6; BlAmyL: *Bacillus licheniformis* alpha-amylase; hEPO: human erythropoietin; hLIF: human leukemia inhibitory factor; AoTAA: *Aspergillus oryzae* alpha-amylase; KmPGU1: *Kluyveromyces marxianus* polygalacturonase; hAZGP1: human zinc-binding alpha-2-glycoprotein; and SfGLU1: *Saccharomycopsis fibuligera* glucoamylase. Data were shown as in Figure 2.

In previous studies on heterologous secretory protein production, endogenous signal sequences were often replaced with ones derived from a host organism. We had showed that in *K. marxianus*, the C-terminal E (or P) in the signal sequence determined the boundary of the hydrophobic core (Figure 4b). Based on this result, we replaced the GLuc signal sequence to the 16th E with heterologous or homologous signal sequences, from the start codon to the hydrophobic stretch followed by the C-terminal E (or P), and measured consequent activity (Figure 6). A fungal amylase from *Aspergillus oryzae* (AoTAA), a yeast polygalacturonase from host *Kluyveromyces marxianus* (KmPGU1), a yeast glucoamylase from *Saccharomycopsis fibuligera* (SfGLU1), and a prokaryotic *Bacillus licheniformis* amylase (BlAmyL) were selected. Of human origin, signal sequences of interleukin 6 (hIL6), erythropoietin (hEPO), leukemia inhibitory factor (hLIF), and alpha-2-glycoprotein 1 (hAZGP1) were selected. Activities of these yGLuc constructs showed extensive variation even though all were recognized as signal sequences (Figure 6). hIL6, BlAmyL, hEPO, and hLIF showed weaker activities than did yGLuc. On the other hand, AoTAA, KmPGU1, hAZGP1, and SfGLU1 showed much stronger activities. It should be noted that the signal sequence of KmPGU1 was derived from the same host organism *K. marxianus*, however, the signal sequence of yeast SfGLU1 from different species exhibited higher activities than did KmPGU1.

Figure 6 shows that a P can be observed in the putative hydrophobic core in hIL6 (11th P), hEPO (7th P), hLIF (10th and 24th P), and hAZGP1 (6th P); all of these constructs showed relatively lower activities, except for hAZGP1. The signal sequence of hAZGP1 has hydrophobic core consisting of VLLSLLLLLG after the 6th P, which seemed not to be deleterious to the overall core hydrophobicity in this case. BlAmyL appeared to have an ideal hydrophobic core, but showed low activity. This sequence contains two Ks and two Rs at the N-terminus, a feature not observed in other eukaryotic signal sequences. AoTAA, KmPGU1, and SfGLU1 contained an N-terminal W, N, and K, respectively, which have a specific role as the N-terminal amino acid before the hydrophobic core (Figure 2b); followed by relatively long stretches of hydrophobic amino acids. These results suggest that there exists an ideal structure for efficient protein secretion in *K. marxianus*.

### Secretory production of the hLIF protein in *K. marxianus*

**Figure 7:**
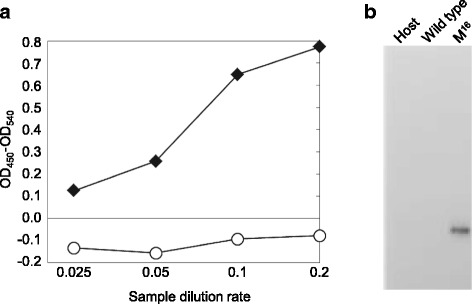
**Addition of the artificial signal sequence M** ^**16**^ **to hLIF increased secretory production in** ***K. marxianus*** **. a)** Expression of constructs containing wild type signal sequence did not show any reaction in an ELISA assay (open circle), but the supernatants from constructs containing M^16^-hLIF:FLAG showed a positive reaction (closed diamonds). **b)** Western blotting of the host strain (RAK3908; Host), a wild type hLIF:FLAG transformant (RAK10252; Wild type), and an M^16^-hLIF:FLAG transformant (RAK11616; M^16^) indicated that only the M^16^-hLIF:FLAG protein was secreted.

The human signal sequences from hIL6, hEPO, and hLIF were not effective for secretory production in *K. marxianus*. To observe the secretory production of human secretory proteins in yeast, we compared expression of the hLIF protein itself, with its original signal sequence and with a version containing the synthetic poly-M^16^ signal sequence (M^16^). The ELISA reactions following the same dilution series of culture supernatants and using an anti-LIF antibody showed that only the M^16^-hLIF:FLAG construct exhibited reaction (Figure 7a). The same supernatants were used for western blotting using an anti-FLAG antibody (Figure 7b). The FLAG antibody reacted with the supernatant from cultures expressing the M^16^-hLIF:FLAG construct but not with the supernatant from wild type-LIF:FLAG construct. These results confirmed again that the M^16^ signal sequence can function efficiently for the production of secretory proteins in the yeast *K. marxianus*.

## Discussion

### Mutational analysis of the yGLuc signal sequence using *K. marxianus* NHEJ cloning

Site-specific mutagenesis is generally conducted through the construction of mutagenized sequences on a vector plasmid in *E. coli*. The plasmid clones must be sequenced because incorrect plasmids are of no use to the subsequent study. Therefore, high-throughput analysis becomes difficult if it contains time-consuming *E. coli* plasmid cloning and sequencing processes. In this study, however, we applied a *K. marxianus* NHEJ cloning system [22] to the construction and analysis of numerous signal sequence mutants. *K. marxianus* exhibits highly efficient NHEJ, such that the ends of introduced fragments are joined efficiently. The construct pKM152 (Figure 1a) contained a *K. marxianus* autonomously replicating sequence KmARS7 and a centromere KmCenD to insure stable plasmid maintenance. Using the primers for deletion of the GLuc signal sequence region and the primers for substitution of the signal sequence regions with artificial amino acid sequences, amplified PCR fragments were used directly for the transformation of *K. marxianus*, where they underwent NHEJ to generate stable plasmid constructs. The resulting transformant colonies were subjected to the luciferase assay to measure the level of luciferase protein secretion. In our case, sequence verification was not conducted except for specific constructs as shown in Table 1 (marked by ^a^); therefore, constructed plasmids might include unexpected mutations. However, we gave priority to comprehensive analysis over the potential inclusion of data from incorrect sequence clones.

**Table 1:** **Yeast strains used in this study**

**Strain name**	**Genotype (** ***K. marxianus*** **except for RAK5125)**	**References**
RAK3908	*ura3-1 ade2-1*	[23]
RAK5125	*S. cerevisiae*: *MAT* **a** *ade2*Δ*0A his3Δ1 leu2Δ0 met15Δ0 ura3Δ0::*Sc*TDH3*p*-*yGLuc_15C_ *LEU2*	[24]
RAK6140	*ura3-1 ade2-1* pKM030 [KmCenD-Sc*ADE2*-KmARS7-Sc*URA3*]	[24]
RAK6205	*ura-13 ade2-1* pKM152 [Sc*TDH3*p-yGLuc-Sc*URA3*-KmCenD-Sc*ADE2*-KmARS7]	This study
RAK8772	*ura3-1 ade2-1* pKM198 [Sc*TDH3*p-M^16^:yGLuc]^a^	This study
RAK9383	*ura3-1 ade2-1* pKM207 [Sc*TDH3*p-F^13^:yGLuc]^a^	This study
RAK9817	*ura3-1 ade2-1* pKM288 [Sc*ADE2*-Sc*TDH3*p-PhEG-Sc*URA3*-KmCenD-KmARS7]	[24]
RAK10336	*ura3-1 ade2-1* pKM414 [Sc*TDH3*p-L^13^:yGLuc]^a^	This study
RAK10339	*ura3-1 ade2-1* pKM417 [Sc*TDH3*p-I^13^:yGLuc]^a^	This study
RAK10252	*ura3-1 ade2-1* pKM398 [Sc*TDH3*p-hLIF:FLAG]^a^	This study
RAK11616	*ura3-1 ade2-1* pKM623 [Sc*TDH3*p-M^16^:hLIF:FLAG]^a^	This study

^a^The signal sequences were verified by DNA sequencing.

To determine the frequency of aberrant mutant clones in the transformant colonies, three clones each from M^16^, F^13^, and L^13^ transformants, and four clones of I^13^ constructs were sequenced. Of the 13 clones, only one, from the I^13^ clones, showed a mutation; this was a deletion of three nucleotides coding for one Ile residue, resulting in an incorrect (I^12^) clone. From this result, we estimated that 12 of 13 clones (92%) would have correct sequence. It may be argued that incorrect clones should not be allowed in the analysis; however, over 90% were accurate clones when sequenced. Also, transformants that showed null value were 17% in the constructs over 0.1-fold (Figure 1b, n/N), suggesting that NHEJ accuracy after PCR amplification is approximately 83-90%. Therefore, we performed direct analysis of transformant colonies without sequence verification.

### The role of specific amino acids in a signal sequence

The comprehensive deletion analysis of signal sequences in this study demonstrated that deletion of the 4th K, or of one or several amino acids in the hydrophobic core, severely decreased the secretory activities of the signal sequences (Figure 1b). These results confirmed the importance of the N-terminal basic amino acid and the hydrophobic core, as previously reported [25-27]. This result also indicated that the hydrophobic core of the yGLuc signal sequence is the minimum required for functioning in *K. marxianus*, because only a single amino acid deletion in the hydrophobic core caused defects of secretory production. Additionally, when the 16th E was deleted (E16Δ), the activity of secreted luciferase increased 4.5-fold, suggesting that the increased hydrophobicity of the core caused by the deletion of a strong hydrophilic amino acid led to enhanced secretory production, because the 17th amino acid is A (see Figure 1). This result is reminiscent of the simple concept that a hydrophobic core is defined as being between hydrophilic amino acids; an N-terminal K and a C-terminal E in this case. This interpretation can be supported by the substitution experiment wherein the 16th E in the GLuc signal sequence was substituted to each of the other amino acids (Figure 2a). Substitution of E16 to L, M, C, F, A, W, V, S, G, Y, H (His), I, Q, or T increased the activity more than two-fold. These amino acids are more hydrophobic than E. In contrast, substitution of the 16th E to D and P decreased the activity, supporting the conclusion of the interference of hydrophilic amino acid in the hydrophobic core and also suggesting a damaging effect of P on a hydrophobic core structure.

The substitution experiment of the N-terminal K to each of the other amino acids indicated that R, N, W, and F can be used instead of K for efficient protein secretion. For these amino acids, however, the N acted as an interfering amino acid when located at the C terminus of a hydrophobic core, similar to the effect of K and R (Figure 2a). N can therefore act as an important amino acid for the constitution of a signal sequence. That W had a positive effect was unexpected; however, the large size of its side chain might provide functionality instead of its basic amino acid structure.

### Modeling the hydrophobic core

To generate a simplified signal sequence, we substituted a part of the yGLuc hydrophobic core with repeats of a single amino acid. The VLFALICI sequence was initially substituted to contain an eight residue repeat of a single amino acid (Figure 3). L^8^, M^8^, W^8^, and F^8^ increased secreted protein activity but repeats containing other residues (I, T, S, Q, Y, A, V, and C) did not. This result indicated that a complex amino acid sequence such as VLFALICI can be substituted with a repeat of select, single amino acids. Furthermore, although eight residue repeats of the residues I, T, S, Q, Y, A, V, and C, appeared not to be suitable, or too weak for a hydrophobic core; in fact, a hydrophobic core consisting of I^12^ and I^13^ was able to function as a signal sequence (Figure 5a). Therefore, the hydrophobic core in a signal sequence can be determined by the number of hydrophobic amino acids without including charged amino acids such as E, D, R, and K. Moreover, the efficiency of secretory production can be determined by the specific repeat number of single hydrophobic amino acid (Figures 4a and 5a). The amino acid L required eleven or twelve repeats for the best production but more than this led to decreased activities. Substitution experiment of human lysozyme signal sequence to poly-L was previously reported and optimum length was presented as L10 when located between N-terminal R and C-terminal P in *S. cerevisiae* [28]. It may be possible that each yeast species has proper length of hydrophobic core. Other amino acids, such as I, F, and M, showed peak activities at the different numbers of repeats. The exclusion of charged amino acids from optimal hydrophobic-core functioning is also supported by the amino acid substitution experiment at the E following the L^13^ hydrophobic core sequence (Figure 4b). The substitution of the C-terminal E of the L^13^ hydrophobic core sequence to any other amino acid decreased the overall activity, indicating that E is the most effective at that site for the determination of hydrophobicity. In other words, it acts as a boundary amino acid for the hydrophobic core. P also functions in this way. All other hydrophobic amino acids, such as G, W, I, L, A, V, C, M, F, and Y, showed much lower activities when placed at this site. Therefore, increased hydrophobicity over an optimal hydrophobic core (as occurred following the latter substitutions) was harmful for signal sequence function.

Secretory production was unexpectedly much higher in hydrophobic cores containing optimized numbers of poly-F and poly-M sequences (Figure 5a). In particular, the activity produced by M^16^ was twenty-four-fold higher than that from the wild type GLuc signal sequence. RT-PCR analysis indicated that the transcriptional levels, in contrast, were all similar to those of the wild type (Figure 5b). Therefore, we found in this study that an artificial poly-M hydrophobic core constituted a supreme signal sequence in the yeast *K. marxianus*. Western blotting of the GLuc revealed that the artificial M^16^ signal sequence enhanced secretion of GLuc protein (Figure 5c). The artificial signal sequence M^16^ can be used as an efficient signal sequence for heterologous protein secretion, as shown with the human LIF protein (Figure 7). We also compared the effect of artificial signal sequences (F^13^, L^13^, and M^16^) with the signal sequences of GLuc and α mating factor (Sc*MFα1*) in the yeast *Saccharomyces cerevisiae* (Additional file 1: Data S1). The signal sequence consisting of F^13^, L^13^, or M^16^ showed higher activity than the wild type, and the M^16^ signal was the best among the synthetic signals examined in *S. cerevisiae*. However, the α-factor signal sequence of *S. cerevisiae* showed higher activity than the M^16^. For optimal secretion, the number of M may need to be adjusted in different yeast species.

At present, we cannot explain why the signal sequence consisting of poly-M is higher than other amino acids. One hypothesis is that poly-M is resistant to degradation pathways. Hydrophobicity of methionine is lower than other hydrophobic amino acids such as Leu, Ile and Phe. The signal consisting of poly-M is in totally enough hydrophobicity to work as a secretion signal but it might not be recognized as a degradation target. Further study is required to understand the effect of poly-M signal sequence on secretion.

### Interpretation of heterologous signal sequences

The optimal signal sequence in *K. marxianus* can be interpreted as follows: the presence of an N-terminal K, R, W, or N; a subsequent hydrophobic core containing an adequate number of non-charged amino acids; and the presence of a C-terminal E or P residue. The validity of this type of signal sequence was examined using heterologous signal sequences (Figure 6). Heterologous signal sequences from proteins across multiple species were attached to the 16th E position using an authentic E or P residue from their original signal sequences. Signal sequences from hIL6, BlAmyL, hEPO, and hLIF showed lower activities but those from AoTAA, KmPGU1, hAZGP1, and SfGLU1 were higher. hIL6 contained an 11th P in its hydrophobic core, which may be the reason for lower secretion in *K. marxianus*. BlAmyL seems to contain an adequate signal sequence if the region from the 10th R to the 23th P was used as the core sequence, but it also contained an additional K and R at the N-terminus, which may play a negative role in *K. marxianus*. hEPO and hLIF contained additional W and K residues, which may play N-terminus roles in front of a hydrophobic core region; however, the hydrophobic sequences following the P residue are too short for optimal function in *K. marxianus*. The AoTAA, KmPGU1, and SfGLU1 sequences have adequate numbers of hydrophobic amino acids following W, N, or K. Among them, SfGLU1 contained the highest number of hydrophobic amino acids without any charged amino acids. We supposed that this was the reason for the efficient secretion driven by the SfGLU1 signal sequence in *K. marxianus*. Together, these results indicated that the length of the non-charged amino acids between the N-terminal W, N, R, or K and the C-terminal P or E determined the efficiency of secretion in *K. marxianus*. The inverse of this finding is that different organisms may have their own N-terminal and C-terminal boundary amino acids to define their hydrophobic core and provide their core sequences with suitable hydrophobicity for optimum secretion.

### N-terminal poly-M proteins

We found that an artificial poly-M sequence can be used as a functional signal sequence in yeast. Therefore, we searched for poly-M sequences in the protein database. Interestingly, N-terminal poly-M containing protein sequences were identified in pathogenic parasites, although all were hypothetical proteins (Additional file 2: Figure S2). These include the CCD59747 protein from *Schistosoma mansoni*, which is a trematode parasite that causes schistosomiasis; EJD73276 from *Loa loa*, which is the filarial nematode that causes Loa loa filariasis; and CDI74732 from *Eimeria praecox*, which is an apicomplexan parasite capable of causing the disease coccidiosis in animals. Based on their relatively unique shared peptide sequence, these N-terminal poly-M proteins may have similar roles in secretion or function in their respective parasitic life cycles.

## Conclusion

The deletion and substitution analyses of the GLuc signal sequence indicated the importance of 4th K and 16th E for determining the length of hydrophobic stretch required for efficient secretory activity. This was a key finding to achieve the following synthetic analysis. We found that the synthetic signal sequences consisting of the N-terminal K, a repeat of a single hydrophobic amino acid such as poly-M, L, I, or F, and the C-terminal E functioned as secretion signals in *K. marxianus* in length-dependent manner. The most efficient synthetic secretion signal was MKM^16^E and it secreted hLIF protein successfully in *K. marxianus* though the native signal sequence of hLIF did not. Based on all the mutational and synthetic analyses, we propose a simple concept of secretory signal sequence in *K. marxianus*, which consists of an N-terminal K, R, W, or N, a subsequent hydrophobic core containing an adequate number of non-charged amino acids, and a C-terminal E or P residue. The model signal sequence structure could explain the secretion activities of GLuc constructs with various secretion signals from human, fungal and bacterial origins.

## Materials and methods

### Yeast strains and growth conditions

Yeast strains used in this study are listed in Table 1. Cells were grown in YPD medium (1% yeast extract, 2% polypeptone, and 2% glucose) or synthetic drop-out media (0.17% yeast nitrogen base without amino acids and ammonium sulfate, 0.5% ammonium sulfate, and 2% glucose and required nutrients) at 28-30°C. Agar (2%) was added to the media if necessary. 5-Fluoroorotic acid (FOA) medium was prepared as described previously [29].

### Polymerase chain reaction (PCR)

Oligonucleotide primers used in this study are listed in Additional file 3: Table S3. The reaction mixture consisted of 5 μl of 2 × KOD FX neo buffer (Toyobo, Osaka, Japan), 2 μl of 2 mM dNTPs, 0.2 μl of KOD FX neo polymerase (Toyobo), and 0.3 μl each of the primer pair (10 μΜ) in a total volume of 10 μl with sterile water. Cycling conditions were as follows: 94°C for 2 min, followed by 30 cycles each of 98°C for 10 s, 65°C for 30 s, and 68°C for 3–4 min. For construction of pKM152, KOD plus polymerase (Toyobo) was used. This reaction mixture consisted of 1 μl of 10 × KOD plus buffer, 1 μl of 2 mM dNTPs, 0.4 μl of 25 mM MgSO_4_, 0.2 μl of KOD plus polymerase, and 0.3 μl each of the primer pair (10 μΜ) in a total volume of 10 μl with sterile water. Cycling conditions were as follows: 94°C for 1 min, followed by 30 cycles each of 94°C for 20 s, 60 or 65°C for 30 s, and 68°C for 1–4 min. The amplified DNA fragments were directly used for yeast transformation.

### Transformation of *K. marxianus*

Transformation of *K. marxianus* was performed as previously described [30]. Briefly, yeast cells (RAK3908) were cultured in 30 ml of YPD medium in a 250 ml baffled flask and shaken at 150 rpm for 24 h at 30°C. The cells were collected by centrifugation and suspended in 900 μl of transformation buffer (TFB), prepared by mixing 20 ml of 60% polyethylene glycol 3350 (Sigma-Aldrich, Tokyo, Japan), 3 ml of 1 M dithiothreitol (Wako, Osaka, Japan), 1.5 ml of 4 M lithium acetate (Kishida Chemical, Osaka, Japan), and 5.5 ml of sterilized water. Next, the cells were centrifuged and resuspended in fresh 600 μl of TFB. Then, 50 μl of the cell suspension was mixed with the amplified DNA fragment (~70 ng) and incubated at 42°C for 30 min. The cell suspension was spread on a synthetic drop-out medium plate and incubated at 28-30°C for 2–3 d.

### Construction of signal sequence mutants of yGLuc

The gene for the *Gaussia princeps* luciferase GLuc (New England BioLabs, Inc., Ipswich, MA, USA) was codon-optimized for yeast expression which was referred to as yGLuc [24]. yGLuc was used in construction of the pKM152 plasmid, and it was maintained in the *K. marxianus* strain RAK6205. The pKM152 contained Sc*TDH3*p-yGLuc (*S. cerevisiae TDH3* promoter-driven yGLuc cassette), Sc*ADE2* and Sc*URA3* selectable markers, an autonomously replicating sequence (KmARS7), and a centromere sequence (KmCenD) (Figure 1a).

pKM152 was generated as follows: the Sc*TDH3*p-yGLuc cassette was amplified from the chromosomal DNA of RAK5125 (*S. cerevisiae*: *MAT***a***ade2*Δ*0A his3Δ1 leu2Δ0 met15Δ0 ura3Δ0::*Sc*TDH3*p*-*yGLuc_15C_*LEU2*) [24] using TDH3-698 and 15G-yGLuc primers. The Sc*URA3* marker gene was amplified from the chromosomal DNA of the *S. cerevisiae* strain BY4704 [23] using 15C-URA3-223 and URA3-300c primers. These two DNA fragments were fused at the 15C:15G annealing sequence using TDH3-698 and URA3+771c primers by fusion PCR [31]. To prepare the vector fragment, a DNA fragment was amplified from the total DNA of RAK6140, which contains the pKM030 plasmid [24], using URA3+771c and URA3+772 primers. Using this amplified DNA fragment as a template, a second DNA fragment was amplified using URA3+772 and KmARS7(201-260)-ADE2-797 primers. For final plasmid construction, the DNA fragment obtained through fusion PCR and the pKM030-derived DNA fragment were mixed and used to transform RAK3908; transformants were selected on uracil drop-out medium. A clone that showed the Ade^+^ Ura^+^ FOA^+^ and Gluc^+^ phenotype was chosen and stocked as RAK6205, containing the pKM152 plasmid.

The construction of mutated yGLuc signal sequences was performed by PCR using KOD FX neo polymerase with total DNA from RAK6205, or using a PCR-amplified pKM152 fragment as a template. Primer pairs utilized are listed in Additional file 4: Table S4. *K. marxianus* has an efficient NHEJ ability [30], which allows the generation of a circular plasmid by joining of the DNA fragment ends [22]. Transformants were selected on adenine drop-out plates. Transformant colonies were picked using toothpicks, inoculated into 96-well plates (TPP Techno Plastic Products AG, Trasadingen, Switzerland) containing 160 μl of uracil drop-out medium, and incubated at 28-30°C for 2 d. Subsequently, 10 μl of the cell culture was inoculated into 290 μl of YPD medium in 96-well plates and incubated at 28-30°C for 1 d. The final culture fluid was used directly for the GLuc luciferase assay.

### GLuc luciferase assay

To measure the luminescence of secreted GLuc proteins, we utilized the BioLux Gaussia Luciferase Assay Kit (New England Biolabs, Inc.). A small aliquot of culture fluid (10 μl) was transferred to a black 96-well microplate (Greiner Bio-One, Frichenhausen, Germany) and mixed with 20 μl of substrate solution. The mixture was incubated for 5 sec, and then subjected to a 1 sec measurement using a Centro LB960 microplate reader (Berthold, Wildbad, Germany). The yeast cell concentration was determined by optical density (OD_600_) using a Power Wave XS microplate reader (BioTek, Winooski, VT, USA). GLuc luciferase activity was expressed in relative luminescence units (RLU = value/(μl · sec · OD_600_)). The fold change was calculated as the ratio of mutagenized GLuc activity per wild type activity. Over twelve colonies were picked for each construct and used for the GLuc assay. Usually, several colonies in each group showed no luciferase activity, probably due to improper plasmid construction. From the blank (negative control: no GLuc insertion) strain measurements, the level of background activity was 0.44 ± 0.20 (average RLU ± standard deviation). Therefore, when the GLuc RLU values of a transformant were not over 0.64, the data were considered to be of null value and were not included in the RLU calculations. In several cases, almost all of the colonies of particular mutant constructs showed null values. It is possible that these designed signal sequence mutations were not functional, but these experiments were not used for calculation because the mutant sequences in these transformants were not confirmed by DNA sequencing. All values except for these null values were subjected to average and standard error calculations.

### Reverse transcription PCR (RT-PCR)

For transcription analysis, total RNA was extracted from the cells of the RAK6205, RAK8772, RAK9383, and RAK10336 strains. The yeast cells were inoculated to 1 ml of uracil drop-out (−U) medium in a 24-well microplate and incubated for 2 d with shaking at 150 rpm at 28°C. An aliquot of the culture (10 μl) was inoculated into 1 ml of YPD medium in a 24-well plate and incubated for 24 h with shaking at 28°C. The yeast cells were collected by centrifugation at 1,000 g for 5 min, suspended in 2 ml of buffer Y (0.1 M EDTA, 1 M sorbitol, 0.7% 2-mercaptoethanol, and 2 mg/ml Zymolyase, pH 7.4), and incubated at 30°C for 30 min. For RNA extraction, the Maxwell 16 LEV simplyRNA Tissue kit (Promega, Tokyo, Japan) was used following the manufacturer’s protocol, and the cell suspension processed in an automated Maxwell 16 Research System (Promega).

DNA (genomic and construct) was removed from the extracted RNA using the Turbo DNA-free kit according to the manufacturer’s protocol (Life Technologies, CA, USA). For reverse transcription, the SuperScript First-Stand Synthesis System for RT-PCR kit (Life Technologies) was used according to the manufacturer’s instructions. DNA-free RNA (100 ng) was used for RT-PCR, and A small fraction (1/50th; 0.5 μl) of the reverse transcribed DNA was used as a template for KOD FX neo PCR in a total of 10 μl using yGLuc+82 and 3CG9-yGLuc+558c primers. Primers KmACT1+16 and KmACT+1111c were used as a control.

### DNA manipulations of the human LIF gene

A human LIF (hLIF) cDNA fragment was amplified from pCAGGS-LIF [32,33] by PCR using the hLIF+1 and hLIF+694c primers. Addition of a FLAG tag (DYKDDDDK) to the C terminus of hLIF was performed by PCR amplification of the hLIF cDNA fragment using the hLIF+1 and 3CG9-FLAGc-hLIF+609c primers. This DNA fragment in turn was used as a template for PCR amplification using the hLIF+1 and URA3+772term3CG9 primers to construct an insert fragment. The vector backbone fragment was prepared by PCR amplification of *K. marxianus* RAK9817 total DNA, which contains the pKM288 plasmid [24], using the URA3+771c and ScTDH3-1c40 primers. These two DNA fragments were mixed and used for transformation of the *K. marxianus* RAK3908 strain. The transformants were selected on –U plates. The sequence of an Ade^+^ Ura^+^ clone was verified and the clone was stored as RAK10252, containing the vector pKM398 (Sc*TDH3*p-hLIF:FLAG).

To attach an artificial signal sequence to the N terminus of the hLIF:Flag construct, template DNA was prepared by PCR amplification of total DNA from the RAK10252 strain using the TDH3p-1c40 and hLIF+4 primers. This DNA fragment was diluted and used as a template for a second PCR using the primer pair MKM(16)Ec-TDH3-1c and hLIF+4. The DNA fragment was used for transformation of the RAK3908 strain; Ade^+^ and Ura^+^ transformants were picked and the sequence of the construct was verified. The RAK11616 strain was stocked as the M^16^-hLIF:FLAG expression strain.

### ELISA

Yeast cells of strain RAK3908 were cultured in 2 ml of YPD, and cells of RAK10252 and RAK11616 were cultured in 2 ml of −U medium for 2 d at 28°C with shaking at 150 rpm. A 20-μl aliquot of the culture was inoculated into 2 ml of YPD and incubated at 28°C with shaking at 150 rpm overnight. The cell culture was transferred to a microtube and centrifuged by 12,000 rpm for 10 min. The supernatant was used for ELISA and western blotting analyses. For assessment of protein levels by ELISA, the MAXISORP plate (Thermo Fisher Scientific Inc., MA USA) and a mouse monoclonal antibody that detects human antigens (anti-hLIF mAbs: 8 μg/ml, clone 9824, R&D Systems Inc., MN, USA) was used. A 50 μl aliquot of the yeast culture supernatant was added to each well and 50 μl of 0.4 μg/ml biotinylated human LIF goat polyclonal antibodies (BAF250, R&D Systems Inc.) in PBS was added to the wells. For detection, Vectastain ABC standard stain solution (Vector laboratories, CA, USA) was used following to manufacture’s protocol. The OD 450 nm and 540 nm values of the samples were measured by a Synergy MX microplate reader (BioTek). The measured quantity of hLIF protein was expressed as the value of OD_450_ - OD_540_.

### Western blotting analyses

For western blotting analysis of GLuc, yeast cells of RAK6205 and RAK8772 strains were incubated in 2 ml of YPD for 1 d, and 500 μl and 450 μl of the culture supernatants, which corresponded to an equivalent number of cells, respectively, were used. To the supernatants, 1 ml of cold acetone was added and centrifuged at 12,000 rpm for 5 min. The precipitate was dissolved in 20 μl of Laemmli Sample Buffer (Bio-Rad, CA, USA) and incubated at 95°C for 5 min. Five μl was loaded to SDS-PAGE equipped with a Cassette Electrophoresis Unit DPE-1020 (Cosmo-bio, Tokyo, Japan) and a SuperSep Ace gel, (5-20%, Wako). After SDS-PAGE, proteins were transferred onto the PVDF membrane by iBlot western blotting system (Life technologies, CA, USA). For protein detection, 1/5000-diluted anti-GLuc antibody (E8023S, New England Biolabs, Inc.), 1/1000-diluted anti-rabbit IgG-HRP (Jackson ImmunoResearch Inc., PA, USA) and Immunostar Zeta (Wako) were used.

In western blotting analysis of hLIF, a 400 μl of yeast culture supernatant containing hLIF proteins was treated using the Endo H_f_ kit (New England Biolabs, Inc.) for protein deglycosylation according to the manufacturer’s instructions. The deglycosylated sample was mixed with 500 μl of cold acetone and the precipitate was collected by centrifugation at 12,000 rpm for 10 min. The precipitate was dissolved in 40 μl of Laemmli Sample Buffer (Bio-Rad) and boiled for 10 min. After SDS-PAGE, proteins were transferred onto the Immobilon PVDF membrane. For protein detection, 1/1000-diluted anti-FLAG monoclonal antibody (1E6, Wako), 1/1000-diluted anti-mouse IgG-HRP (Jackson ImmunoResearch), and Immunostar Zeta (Wako) were used.

## Additional files

Additional file 1: Data S1. Secretion ability of the synthetic signal sequences in the yeast *Saccharomyces cerevisiae*. Additional file 2: Figure S2. N-terminal poly-M containing proteins identified in protein database. These are sequences from pathogenic parasites. All are hypothetical proteins. *Schistosoma mansoni*, which is a trematode parasite that causes schistosomiasis; *Loa loa*, which is the filarial nematode that causes Loa loa filariasis; and *Eimeria praecox*, which is an apicomplexan parasite capable of causing the disease coccidiosis in animals. Additional file 3: Table S3. Primers used in this study. Additional file 4: Table S4. Primer pairs for plasmid DNA constructions.

## Abbreviations

AoTAA: *Aspergillus oryzae* amylase
BlAmyL: *Bacillus licheniformis* amylase
ER: Endoplasmic reticulum
FOA: 5-Fluoroorotic acid
GLuc: *Gaussia princeps* secretory luciferase
hEPO: Human erythropoietin
hIL6: Human interleukin 6
hLIF: Human leukemia inhibitory factor
hAZGP1: Human alpha-2-glycoprotein 1
KmARS7: *Kluyveromyces marxianus* autonomously replicating sequence 7
KmCenD: *K. marxianus* centromere sequence D
KmPGU1: *K. marxianus* polygalacturonase
NHEJ: Non-homologous end joining
PCR: Polymerase chain reaction
RT-PCR: Reverse transcription PCR
Sc*TDH3*p-yGLuc: *Saccharomyces cerevisiae TDH3* promoter-driven yGLuc cassette
SDS-PAGE: Sodium dodecyl sulfate polyaclylamide gel electrophoresis
SfGLU1: *Saccharomycopsis fibuligera* glucoamylase
TFB: Transformation buffer
−U: Uracil drop-out
