# Supplementary material for: Synthetic signal sequences that enable efficient secretory protein production in the yeast Kluyveromyces marxianus
Source: Microb Cell Fact. 2015 Feb 14;14:20. doi: 10.1186/s12934-015-0203-y (PMC4347551; doi:10.1186/s12934-015-0203-y)
Supplement: Additional file 1: Data S1. — Secretion ability of the synthetic signal sequences in the yeast Saccharomyces cerevisiae. [file 12934_2015_203_MOESM1_ESM.pdf]

Data S1 Secretion ability of the synthetic signal sequences in the yeast *Saccharomyces cerevisiae*.

Table S1-1 Analysis of the signal sequences in the yeast *Saccharomyces cerevisiae*

Name	Signal sequence	$\times 10^5$ RLU±SE	Fold	n/N
Wild type	MGVKVLFALICIAVAE	4.2 ± 0.8	1	24/24
F^{13}	MKFFFFFFFFFFFFFE	48.1 ± 7.8	11	12/12
L^{13}	MKLLLLLLLLLLLLLE	477.0 ± 417.9	112	12/12
M^{16}	MKMMMMMMMMMMMMMMMME	6450.9 ± 1027.9	1513	15/15
ScMFα1	MRFPSIFTAVLFAASSALAAE	10906.3 ± 2317.2	2558	12/12

YEp plasmids containing yGLuc with various signal sequences were constructed in the yeast *Saccharomyces cerevisiae* (see Methods and Tables below). RLU; relative luminescence units (value/(μl•sec•OD600)), Fold; ratio of GLuc activity with artificial signal sequence per wild type activity. n; number of transformants used for calculation, N; number of transformants measured. The luminescence was measured using a GloMax™ 20/20 Luminometer (Promega, Madison, WI, USA).

Methods; Construction of yGLuc plasmids in *S. cerevisiae*

The gap-repair method was used for the plasmid construction in *S. cerevisiae* according to the previously published procedure (Makanae *et al*., 2013). KOD FX neo DNA polymerase (Toyobo) was used for PCR reactions. For the addition of artificial signal sequences of M^{16}, F^{13}, and L^{13} to yGLuc, DNA fragments were produced by PCR using TDH3-572 and 3CG9-yGLuc+558c primers from total DNA of *K. marxianus* strains RAK8772 (M^{16}), RAK9383 (F^{13}), and RAK10336 (L^{13}), respectively,

as templates. The wild type yGLuc was produced using the same primers from total DNA of RAK6205 strain as a template. These PCR products were used as a template for the second PCR using TDH3-572 and ScURA3(717786TAA)-3CG9 primers. The vector DNA was prepared by PCR using URA3+771c and TDH3-1c40 primers from YEpGAP-cherry plasmid DNA (Keppler-Ross *et al.*, 2008) as a template. The second PCR products and the vector DNA were mixed and used for the transformation of *S. cerevisiae* BY4700 strain. The transformants were selected on uracil drop-out plates. For the addition of ScMFα1 signal sequence to yGLuc, the wild type PCR product amplified from total DNA of RAK6205 strain was used as template for the second PCR using ScMFalpha1+13to60-yGLuc+46 and ScURA3(717786TAA)-3CG9 primers. The vector DNA fragment was amplified from YEpGAP-cherry plasmid using URA3+771c and ScTDH3p-1c-ScMFalpha1+1to52c primers. These DNA fragments were mixed and used for the transformation of BY4700.

Table S1-2 *Saccharomyces cerevisiae* strains in Supplementary Data S1

Strain	Genotype
BY4700	*MAT***a** *ura3Δ0*
RAK11908	*MAT***a** *ura3Δ0* YEp-Sc*TDH3*p-yGLuc-Sc*URA3*
RAK11911	*MAT***a** *ura3Δ0* YEp-Sc*TDH3*p-M^{16}:yGLuc-Sc*URA3*
RAK11914	*MAT***a** *ura3Δ0* YEp-Sc*TDH3*p-F^{13}:yGLuc-Sc*URA3*
RAK11917	*MAT***a** *ura3Δ0* YEp-Sc*TDH3*p-L^{13}:yGLuc-Sc*URA3*
RAK13106	*MAT***a** *ura3Δ0* YEp-Sc*TDH3*p-Sc*MFα1*(1-60):yGLuc-Sc*URA3*

Table S1-3 Primers used for *S. cerevisiae* plasmid constructions in Supplementary Data S1

Primer name	Sequence (5' - 3')
TDH3-572	gctgtaacccgtacatgcccaaaat
3CG9-yGLuc+558c	cccgggcccttagtcaccaccagcacccttgatc
ScURA3(717786TAA)-3CG9	aaagggaagggatgctaaggtagagggtgaacgttacagaaaagc aggctgggaagcatatttgagaagataacccgggccc
URA3+771c	ttcccagcctgcttttctgtaacgt
TDH3-1c40	tttgtttgtttatgtgtgtttattcgaaactaagttcttg
ScMFalpha1+13to60-yGLuc+46	tcaatttttactgcagttttattcgcagcatcctccgcattagctgctgaa gctaagccaaccgaaaaca
ScTDH3p-1c-ScMFalpha1+1to52 c	atgcggaggatgctgcgaataaaactgcagtaaaaattgaaggaaat ctcattttgtttgtttatgtgtg

References for Supplementary Data S1

Makanae K, Kintaka R, Makino T, Kitano H, Moriya H: Identification of dosage-sensitive genes in *Saccharomyces cerevisiae* using the genetic tug-of-war method. Genome Res 2013, 23:300-311.

Keppler-Ross S, Noffz C, Dean N: A new purple fluorescent color marker for genetic studies in *Saccharomyces cerevisiae* and *Candida albicans*. Genetics 2008, 179:705-710.
