# Supplementary material for: Synthetic signal sequences that enable efficient secretory protein production in the yeast Kluyveromyces marxianus
Source: Microb Cell Fact. 2015 Feb 14;14:20. doi: 10.1186/s12934-015-0203-y (PMC4347551; doi:10.1186/s12934-015-0203-y)
Supplement: Additional file 2: Figure S2. — N-terminal poly-M containing proteins identified in protein database. These are sequences from pathogenic parasites. All are hypothetical proteins. Schistosoma mansoni, which is a trematode parasite that causes schistosomiasis; Loa loa, which is the filarial nematode that causes Loa loa filariasis; and Eimeria praecox, which is an apicomplexan parasite capable of causing the disease coccidiosis in animals. [file 12934_2015_203_MOESM2_ESM.pdf]

Schistosoma mansoni<br>CCD59747	MMMMMMMMMMMMMMMMMMMMMMMIMLIIITSIIHIIVMMGSVISITDKDSVIVSYSW
Loa loa<br>EJD73267	MMMMMMMMMMMMMMMMMMMMMMMMMMMMMMMMMMMMTMMKIEFPHFLVQHLITLWLAMLMEVSKFSLYHLHL
Eimeria praecox<br>CDI74732	MMMMMMMMMMMMMMMMMMMLLLLLLVMVMVMVMVMVVLLLLLLLLLLLVILVVVVLLLLLLLLLLLLLLLLL<br>VMVMVMVMVMVVVVMVMKEEVSRLRTQLDKQRNKNAMLKQRLEQLQDKLKELELASAKPCTEDSPEIRSMRI<br>IEGRSGKQQQQQQQQQQQQQQQQQQQEHPVS

Figure S2

N-terminal poly-M containing proteins identified in protein database. These are sequences from pathogenic parasites. All are hypothetical proteins. *Schistosoma mansoni*, which is a trematode parasite that causes schistosomiasis; *Loa loa*, which is the filarial nematode that causes Loa loa filariasis; and *Eimeria praecox*, which is an apicomplexan parasite capable of causing the disease coccidiosis in animals.
