# Supplementary material for: Synthetic signal sequences that enable efficient secretory protein production in the yeast Kluyveromyces marxianus
Source: Microb Cell Fact. 2015 Feb 14;14:20. doi: 10.1186/s12934-015-0203-y (PMC4347551; doi:10.1186/s12934-015-0203-y)
Supplement: Additional file 3: Table S3. — Primers used in this study. [file 12934_2015_203_MOESM3_ESM.pdf]

Table S3 Primers used in this study

Primer name	Sequence (5'-3')
TDH3-698	ataaaaaacacgctttttcagttcg
15G-yGLuc	gggggggggggggggttagtcaccaccagcacc
15C-URA3-223	cccccccccccccccaagcttttcaattcatcttttttttttttg
URA3-300c	tgttgtgaagtcattgacacag
KmARS7(201-260)-ADE2-797	caagacttcttgaagtgaaaaccaactttcagtcttcaaactaaaaatgaaaatcagtggcgctggcgcatctgttcct
URA3+771c	ttcccagcctgcttttctgtaacgt
URA3+772	gcatatttgagaagatgcggccagc
TDH3-1c40	tttgtttgtttatgtgtgtttattcgaaactaagttcttg
yGLuc+24c	agcgaacaagaccttgacacccat
yGLuc+27c	caaagcgaacaagaccttgacaccc
yGLuc+30c	gatcaaagcgaacaagaccttgaca
yGLuc+33c	acagatcaaagcgaacaagaccttg
yGLuc+36c	gatacagatcaaagcgaacaagacc
yGLuc+39c	agcgatacagatcaaagcgaacaag
yGLuc+42c	gacagcgatacagatcaaagcgaac
yGLuc+45c	agcgacagcgatacagatcaaagcg
yGLuc+48c	ttcagcgacagcgatacagatcaaa
yGLuc+51c	agcttcagcgacagcgatacagatc
yGLuc+54c	cttagcttcagcgacagcgatacag
yGLuc+57c	tggcttagcttcagcgacagcgata
yGLuc+60c	ggttggcttagcttcagcgacagcg
yGLuc+63c	ttcggttggcttagcttcagcgaca
yGLuc+66c	gttttcggttggcttagcttcagcg
yGLuc+69c	gttgttttcggttggcttagcttca
yGLuc+72c	ttcgttgttttcggttggcttagct
yGLuc+75c	gtcttcgttgttttcggttggctta
yGLuc+78c	gaagtcttcgttgttttcggttggc
yGLuc+81c	gttgaagtcttcgttgttttcggtt
yGLuc+90c	agcgacgatgttgaagtcttcgttg
yGLuc+108c	agcgaagttagaagcgacagcgacg
yGLuc+12c-TDH3-1c	cttgacacccattttgtttgtttat
yGLuc+3-TDH3-1c	cattttgtttgtttatgtgtgtttattcga
yGLuc+4	ggtgtcaaggtcttgttcgctttga
yGLuc+7	gtcaaggtcttgttcgctttgatct
yGLuc+10	aaggtcttgttcgctttgatctgta
yGLuc+13	gtcttgttcgctttgatctgtatcg
yGLuc+16	ttgttcgctttgatctgtatcgctg
yGLuc+19	ttcgctttgatctgtatcgctgtcg

yGLuc+22	gctttgatctgtatcgctgtcgctg
yGLuc+25	ttgatctgtatcgctgtcgctgaag
yGLuc+28	atctgtatcgctgtcgctgaagcta
yGLuc+31	tgtatcgctgtcgctgaagctaagc
yGLuc+34	atcgctgtcgctgaagctaagccaa
yGLuc+37	gctgtcgctgaagctaagccaaccg
yGLuc+40	gtcgctgaagctaagccaaccgaaa
yGLuc+43	gctgaagctaagccaaccgaaaaca
yGLuc+46	gaagctaagccaaccgaaaacaacg
yGLuc+49	gctaagccaaccgaaaacaacgaag
yGLuc+52	aagccaaccgaaaacaacgaagact
yGLuc+55	ccaaccgaaaacaacgaagacttca
yGLuc+58	accgaaaacaacgaagacttcaaca
yGLuc+61	gaaaacaacgaagacttcaacatcg
yGLuc+64	aacaacgaagacttcaacatcgtcg
yGLuc+70	gaagacttcaacatcgtcgctgtcg
yGLuc+73	gacttcaacatcgtcgctgtcgctt
yGLuc+76	ttcaacatcgtcgctgtcgcttcta
yGLuc+79	aacatcgtcgctgtcgcttctaact
yGLuc+82	atcgtcgctgtcgcttctaacttcg
yGLuc+91	gtcgcttctaacttcgctaccaccg
yGLuc+100	aacttcgctaccaccgacttggacg
yGLuc+109	accaccgacttggacgctgacagag
yGLuc+118	ttggacgctgacagaggtaagttgc
Fc-yGLuc+45c	gaaagcgacagcgatacagatcaaagcg
Lc-yGLuc+45c	caaagcgacagcgatacagatcaaagcg
Ic-yGLuc+45c	gatagcgacagcgatacagatcaaagcg
Mc-yGLuc+45c	catagcgacagcgatacagatcaaagcg
Vc-yGLuc+45c	aacagcgacagcgatacagatcaaagcg
Sc-yGLuc+45c	ggaagcgacagcgatacagatcaaagcg
Pc-yGLuc+45c	tggagcgacagcgatacagatcaaagcg
Tc-yGLuc+45c	ggtagcgacagcgatacagatcaaagcg
Ac-yGLuc+45c	agcagcgacagcgatacagatcaaagcg
Yc-yGLuc+45c	gtaagcgacagcgatacagatcaaagcg
Hc-yGLuc+45c	gtgagcgacagcgatacagatcaaagcg
Qc-yGLuc+45c	ttgagcgacagcgatacagatcaaagcg
Nc-yGLuc+45c	gttagcgacagcgatacagatcaaagcg
Kc-yGLuc+45c	cttagcgacagcgatacagatcaaagcg
Dc-yGLuc+45c	gtcagcgacagcgatacagatcaaagcg
Cc-yGLuc+45c	acaagcgacagcgatacagatcaaagcg
Wc-yGLuc+45c	ccaagcgacagcgatacagatcaaagcg

Rc-yGLuc+45c	tctagcgacagcgatacagatcaaagcg
Gc-yGLuc+45c	accagcgacagcgatacagatcaaagcg
MFc-TDH3-1c	gaacattttgtttgtttatgtgtgtttattcga
MLc-TDH3-1c	caacattttgtttgtttatgtgtgtttattcga
MIc-TDH3-1c	gatcattttgtttgtttatgtgtgtttattcga
MMc-TDH3-1c	catcattttgtttgtttatgtgtgtttattcga
MVc-TDH3-1c	aaccattttgtttgtttatgtgtgtttattcga
MSc-TDH3-1c	ggacattttgtttgtttatgtgtgtttattcga
MPc-TDH3-1c	tggcattttgtttgtttatgtgtgtttattcga
MTc-TDH3-1c	ggtcattttgtttgtttatgtgtgtttattcga
MAc-TDH3-1c	agccattttgtttgtttatgtgtgtttattcga
MYc-TDH3-1c	gtacattttgtttgtttatgtgtgtttattcga
MHc-TDH3-1c	gtgcattttgtttgtttatgtgtgtttattcga
MQc-TDH3-1c	ttgcattttgtttgtttatgtgtgtttattcga
MNc-TDH3-1c	gttcattttgtttgtttatgtgtgtttattcga
MDc-TDH3-1c	gtccattttgtttgtttatgtgtgtttattcga
MEc-TDH3-1c	ttccattttgtttgtttatgtgtgtttattcga
MCc-TDH3-1c	acacattttgtttgtttatgtgtgtttattcga
MWc-TDH3-1c	ccacattttgtttgtttatgtgtgtttattcga
MRc-TDH3-1c	tctcattttgtttgtttatgtgtgtttattcga
MGc-TDH3-1c	acccattttgtttgtttatgtgtgtttattcga
MKL8-hGLuc+37	atgaaactgctgctgctgctgctgctgctggctgtggccgaggccaagcccaccgagaacaacgaagacttcaacatcgt
MRL8-hGLuc+37	atgaggctgctgctgctgctgctgctgctggctgtggccgaggccaagcccaccgagaacaacgaagacttcaacatcgt
MRM8-hGLuc+37	atgaggatgatgatgatgatgatgatgatggctgtggccgaggccaagcccaccgagaacaacgaagacttcaacatcgt
MRC8-hGLuc+37	atgaggtgctgctgctgctgctgctgctgcgctgtggccgaggccaagcccaccgagaacaacgaagacttcaacatcgt
MRF8-hGLuc+37	atgaggttcttcttcttcttcttcttcttcgctgtggccgaggccaagcccaccgagaacaacgaagacttcaacatcgt
MRA8-hGLuc+37	atgagggccgccgccgccgccgccgccgccgctgtggccgaggccaagcccaccgagaacaacgaagacttcaacatcgt
MRW8-hGLuc+37	atgaggtggtggtggtggtggtggtggtgggctgtggccgaggccaagcccaccgagaacaacgaagacttcaacatcgt
MRV8-hGLuc+37	atgagggtggtggtggtggtggtggtggtggctgtggccgaggccaagcccaccgagaacaacgaagacttcaacatcgt
MRY8-hGLuc+37	atgaggtactactactactactactactacgctgtggccgaggccaagcccaccgagaacaacgaagacttcaacatcgt
MRI8-hGLuc+37	atgaggatcatcatcatcatcatcactactgctgtggccgaggccaagcccaccgagaacaacgaagacttcaacatcgt

MRS8-hGLuc+37	atgaggtcctcctcctcctcctcctcctccgctgtggccgaggccaagcccac cgagaacaacgaagacttcaacatcgt
MRT8-hGLuc+37	atgaggaccaccaccaccaccaccaccaccgctgtggccgaggccaagcc caccgagaacaacgaagacttcaacatcgt
MRQ8-hGLuc+37	atgaggcagcagcagcagcagcagcagcaggctgtggccgaggccaagc ccaccgagaacaacgaagacttcaacatcgt
MKL(7)-hGLuc+46	atgaaactgctgctgctgctgctggaggccaagcccaccgagaacaac gaagacttcaacatcgt
MKL(8)-hGLuc+46	atgaaactgctgctgctgctgctgctggaggccaagcccaccgagaaca acgaagacttcaacatcgt
MKL(9)-hGLuc+46	atgaaactgctgctgctgctgctgctgctggaggccaagcccaccgaga acaacgaagacttcaacatcgt
MKL(10)-hGLuc+46	atgaaactgctgctgctgctgctgctgctgctggaggccaagcccaccg agaacaacgaagacttcaacatcgt
MKL(11)-hGLuc+46	atgaaactgctgctgctgctgctgctgctgctgctggaggccaagcccac cgagaacaacgaagacttcaacatcgt
MKL(12)-hGLuc+46	atgaaactgctgctgctgctgctgctgctgctgctgctggaggccaagcc caccgagaacaacgaagacttcaacatcgt
MKL(13)-hGLuc+46	atgaaactgctgctgctgctgctgctgctgctgctgctgctggaggccaa gcccaccgagaacaacgaagacttcaacatcgt
MKL(14)-hGLuc+46	atgaaactgctgctgctgctgctgctgctgctgctgctgctgctggaggcc aagcccaccgagaacaacgaagacttcaacatcgt
MKL(15)-hGLuc+46	atgaaactgctgctgctgctgctgctgctgctgctgctgctgctgctggag gccaagcccaccgagaacaacgaagacttcaacatcgt
MKL(16)-hGLuc+46	atgaaactgctgctgctgctgctgctgctgctgctgctgctgctgctgctg gaggccaagcccaccgagaacaacgaagacttcaacatcgt
MKL(17)-hGLuc+46	atgaaactgctgctgctgctgctgctgctgctgctgctgctgctgctgctgc tggaggccaagcccaccgagaacaacgaagacttcaacatcgt
MKL(13)F-hGLuc+46	atgaaactgctgctgctgctgctgctgctgctgctgctgctgttcgccaag cccaccgagaacaacgaagacttcaacatcgt
MKL(13)L-hGLuc+46	atgaaactgctgctgctgctgctgctgctgctgctgctgctgctggccaag cccaccgagaacaacgaagacttcaacatcgt
MKL(13)I-hGLuc+46	atgaaactgctgctgctgctgctgctgctgctgctgctgctgatcgccaag cccaccgagaacaacgaagacttcaacatcgt
MKL(13)M-hGLuc+46	atgaaactgctgctgctgctgctgctgctgctgctgctgctgatggccaag cccaccgagaacaacgaagacttcaacatcgt
MKL(13)V-hGLuc+46	atgaaactgctgctgctgctgctgctgctgctgctgctgctggtggccaag cccaccgagaacaacgaagacttcaacatcgt
MKL(13)S-hGLuc+46	atgaaactgctgctgctgctgctgctgctgctgctgctgctgtccgccaag cccaccgagaacaacgaagacttcaacatcgt
MKL(13)P-hGLuc+46	atgaaactgctgctgctgctgctgctgctgctgctgctgctgcccgccaag cccaccgagaacaacgaagacttcaacatcgt

MKL(13)T-hGLuc+46	atgaaactgctgctgctgctgctgctgctgctgctgctgctgctgaccgccaagcccaccgagaacaacgaagacttcaacatcgt
MKL(13)A-hGLuc+46	atgaaactgctgctgctgctgctgctgctgctgctgctgctgctggccgccaagcccaccgagaacaacgaagacttcaacatcgt
MKL(13)Y-hGLuc+46	atgaaactgctgctgctgctgctgctgctgctgctgctgctgctgtacgccaagcccaccgagaacaacgaagacttcaacatcgt
MKL(13)H-hGLuc+46	atgaaactgctgctgctgctgctgctgctgctgctgctgctgctgcacgccaagcccaccgagaacaacgaagacttcaacatcgt
MKL(13)Q-hGLuc+46	atgaaactgctgctgctgctgctgctgctgctgctgctgctgctgcaggccaagcccaccgagaacaacgaagacttcaacatcgt
MKL(13)N-hGLuc+46	atgaaactgctgctgctgctgctgctgctgctgctgctgctgctgaacgccaagcccaccgagaacaacgaagacttcaacatcgt
MKL(13)K-hGLuc+46	atgaaactgctgctgctgctgctgctgctgctgctgctgctgctgaaagccaagcccaccgagaacaacgaagacttcaacatcgt
MKL(13)D-hGLuc+46	atgaaactgctgctgctgctgctgctgctgctgctgctgctgctggacgccaagcccaccgagaacaacgaagacttcaacatcgt
MKL(13)C-hGLuc+46	atgaaactgctgctgctgctgctgctgctgctgctgctgctgctgtgcgccaagcccaccgagaacaacgaagacttcaacatcgt
MKL(13)W-hGLuc+46	atgaaactgctgctgctgctgctgctgctgctgctgctgctgctgtgggccaagcccaccgagaacaacgaagacttcaacatcgt
MKL(13)R-hGLuc+46	atgaaactgctgctgctgctgctgctgctgctgctgctgctgctgagggccaagcccaccgagaacaacgaagacttcaacatcgt
MKL(13)G-hGLuc+46	atgaaactgctgctgctgctgctgctgctgctgctgctgctgctgggcgccaagcccaccgagaacaacgaagacttcaacatcgt
MKI(7)-hGLuc+46	atgaaaatcatcatcatcatcatcatcgaggccaagcccaccgagaacaacgaagacttcaacatcgt
MKI(8)-hGLuc+46	atgaaaatcatcatcatcatcatcatcatcgaggccaagcccaccgagaacaacgaagacttcaacatcgt
MKI(9)-hGLuc+46	atgaaaatcatcatcatcatcatcatcatcatcgaggccaagcccaccgagaacaacgaagacttcaacatcgt
MKI(10)-hGLuc+46	atgaaaatcatcatcatcatcatcatcatcatcatcgaggccaagcccaccgagaacaacgaagacttcaacatcgt
MKI(11)-hGLuc+46	atgaaaatcatcatcatcatcatcatcatcatcatcatcgaggccaagcccaccgagaacaacgaagacttcaacatcgt
MKI(12)-hGLuc+46	atgaaaatcatcatcatcatcatcatcatcatcatcatcatcgaggccaagcccaccgagaacaacgaagacttcaacatcgt
MKI(13)-hGLuc+46	atgaaaatcatcatcatcatcatcatcatcatcatcatcatcatcgaggccaagcccaccgagaacaacgaagacttcaacatcgt
MKI(14)-hGLuc+46	atgaaaatcatcatcatcatcatcatcatcatcatcatcatcatcatcgaggccaagcccaccgagaacaacgaagacttcaacatcgt
MKI(15)-hGLuc+46	atgaaaatcatcatcatcatcatcatcatcatcatcatcatcatcatcatcgaggccaagcccaccgagaacaacgaagacttcaacatcgt

MKI(16)-hGLuc+46	atgaaaatcatcatcatcatcatcatcatcatcatcatcatcatcatcatcatcga<br>ggccaagcccaccgagaacaacgaagacttcaacatcgt
MKI(17)-hGLuc+46	atgaaaatcatcatcatcatcatcatcatcatcatcatcatcatcatcatcatcatc<br>gaggccaagcccaccgagaacaacgaagacttcaacatcgt
MKF(7)-hGLuc+46	atgaaattcttcttcttcttcttcttcgaggccaagcccaccgagaacaacgaag<br>acttcaacatcgt
MKF(8)-hGLuc+46	atgaaattcttcttcttcttcttcttcttcgaggccaagcccaccgagaacaacga<br>agacttcaacatcgt
MKF(9)-hGLuc+46	atgaaattcttcttcttcttcttcttcttcttcgaggccaagcccaccgagaacaac<br>gaagacttcaacatcgt
MKF(10)-hGLuc+46	atgaaattcttcttcttcttcttcttcttcttcttcgaggccaagcccaccgagaac<br>aacgaagacttcaacatcgt
MKF(11)-hGLuc+46	atgaaattcttcttcttcttcttcttcttcttcttcttcgaggccaagcccaccgaga<br>acaacgaagacttcaacatcgt
MKF(13)-hGLuc+46	atgaaattcttcttcttcttcttcttcttcttcttcttcttcttcgaggccaagcccacc<br>gagaacaacgaagacttcaacatcgt
MKF(15)-hGLuc+46	atgaaattcttcttcttcttcttcttcttcttcttcttcttcttcttcttcgaggccaagc<br>ccaccgagaacaacgaagacttcaacatcgt
MKF(17)-hGLuc+46	atgaaattcttcttcttcttcttcttcttcttcttcttcttcttcttcttcttcttcgaggcc<br>aagcccaccgagaacaacgaagacttcaacatcgt
MKM(7)-hGLuc+46	atgaaaatgatgatgatgatgatgatggaggccaagcccaccgagaacaac<br>gaagacttcaacatcgt
MKM(8)-hGLuc+46	atgaaaatgatgatgatgatgatgatgatggaggccaagcccaccgagaaca<br>acgaagacttcaacatcgt
MKM(9)-hGLuc+46	atgaaaatgatgatgatgatgatgatgatgatggaggccaagcccaccgaga<br>acaacgaagacttcaacatcgt
MKM(10)-hGLuc+46	atgaaaatgatgatgatgatgatgatgatgatgatggaggccaagcccaccg<br>agaacaacgaagacttcaacatcgt
MKM(11)-hGLuc+46	atgaaaatgatgatgatgatgatgatgatgatgatgatggaggccaagcccac<br>cgagaacaacgaagacttcaacatcgt
MKM(12)-hGLuc+46	atgaaaatgatgatgatgatgatgatgatgatgatgatgatggaggccaagcc<br>caccgagaacaacgaagacttcaacatcgt
MKM(13)-hGLuc+46	atgaaaatgatgatgatgatgatgatgatgatgatgatgatgatggaggccaa<br>gcccaccgagaacaacgaagacttcaacatcgt
MKM(14)-hGLuc+46	atgaaaatgatgatgatgatgatgatgatgatgatgatgatgatgatggaggcc<br>aagcccaccgagaacaacgaagacttcaacatcgt
MKM(15)-hGLuc+46	atgaaaatgatgatgatgatgatgatgatgatgatgatgatgatgatgatggag<br>gccaagcccaccgagaacaacgaagacttcaacatcgt
MKM(16)-hGLuc+46	atgaaaatgatgatgatgatgatgatgatgatgatgatgatgatgatgatgatg<br>gaggccaagcccaccgagaacaacgaagacttcaacatcgt
MKM(17)-hGLuc+46	atgaaaatgatgatgatgatgatgatgatgatgatgatgatgatgatgatgatga<br>tggaggccaagcccaccgagaacaacgaagacttcaacatcgt

TAA+(1-54)-hGLuc+49	atgatggtcgcgtggtggtctctatttctgtacggccttcaggtcgcggcacct gccaagcccaccgagaacaacgaagacttcaacatcgt
SfGLU1+(1-54)-hGLuc+49	atgaaattcggtgttttattttccgtctttgctgctattgttagtgctttacctgccaa gcccaccgagaacaacgaagacttcaacatcgt
AmyLN5C+(1-27)c-TDH3-1c	ggcgtaaagccgtttttgttgtttcattttgtttgtttatgtgtgtttattcga
AmyLN5C+(28-69)-hGLuc+49	cgattgctgacgctgttatttgcgctcatcttcttgctgcctgccaagcccaccg agaacaacgaagacttcaacatcgt
KmPGU1+(1-54)-hGLuc+49	atgttattcagcaacaccttattaatcgcagcagctagtgcattattagctgaag ccaagcccaccgagaacaacgaagacttcaacatcGT
hIL6+(1-33)c-TDH3-1c	tggaccgaaggcacttgtggagaaggagttcattttgtttgtttatgtgtgttta
hIL6+(34-69)-hGLuc+49	gttgccttctccctggggctgctcctggtgttgcctgctaagccaaccgaaaac aacgaag
hEPO+(1-21)c-TDH3-1c	aggacaaccgtgcacccccattttgtttgtttatgtgtgttta
hEPO+(22-60)-yGLuc+49	gcctggctgtggcttctcctgtccctgctgtcgctccctgctaagccaaccgaa aacaacgaag
hLIF+(1-30)c-TDH3−1c	gggcacaactcctgccgccaagaccttcattttgtttgtttatgtgtgtttattcga
hLIF+(31-78)-yGLuc+49	ctgctgttggttctgcactggaaacatggggcggggagccccctccccgcta agccaaccgaaaacaacgaag
hZA2G+(1-18)c-TDH3-1c*	aggcaccattcttaccattttgtttgtttatgtgtgttta
hZA2G+(19-51)-yGLuc+49*	gtcctgctgtctctgctgctgcttctgggtcctgctaagccaaccgaaaacaac gaag
3CG9-yGLuc+558c	cccgggcccttagtcaccaccagcacccttgatc
KmACT1+16	gcagaggtcgctgctttagttattg
KmACT+1111c	atggaccagattcgtcgtattcttg
hLIF+1	atgaaggtcttggcggcaggag
hLIF+694c	gcacctgaggagtgaattcc
3CG9-FLAGc-hLIF+609c	cccgggcccttacttgtcgtcgtcgtccttgtagtcgaaggcctgggccaaca cggcgatg
URA3+772term3CG9	gcatatttgagaagataaaaaactgtattataagtaaatgcatgtatactaaactc acaaattagagcttcaatttaattatatcagttacccgggccc
MKM(16)Ec-TDH3p-1c	ctccatcatcatcatcatcatcatcatcatcatcatcatcatcatcatcattttcattt tgtttgtttatgtgtgtttattcga
hLIF+4	aaggtcttggcggcaggagt

*hZA2G is an alternative name for hAZGP1.
