# Supplementary material for: Synthetic signal sequences that enable efficient secretory protein production in the yeast Kluyveromyces marxianus
Source: Microb Cell Fact. 2015 Feb 14;14:20. doi: 10.1186/s12934-015-0203-y (PMC4347551; doi:10.1186/s12934-015-0203-y)
Supplement: Additional file 4: Table S4. — Primer pairs for plasmid DNA constructions. [file 12934_2015_203_MOESM4_ESM.pdf]

Table S4 Primer pairs for plasmid DNA constructions

Construct name	Primer 1	Primer 2
Wild type	URA3+771c	URA3+772
G2Δ	yGLuc+3-TDH3-1c	yGLuc+7
G2V3Δ	yGLuc+3-TDH3-1c	yGLuc+10
G2-K4Δ	yGLuc+3-TDH3-1c	yGLuc+13
V5Δ	yGLuc+12c-TDH3-1c	yGLuc+16
V5L6Δ	yGLuc+12c-TDH3-1c	yGLuc+19
V5-F7Δ	yGLuc+12c-TDH3-1c	yGLuc+22
V5-A8V	yGLuc+12c-TDH3-1c	yGLuc+25
L9Δ	yGLuc+24c	yGLuc+28
I10Δ	yGLuc+27c	yGLuc+31
C11Δ	yGLuc+30c	yGLuc+34
I12Δ	yGLuc+33c	yGLuc+37
A13Δ	yGLuc+36c	yGLuc+40
V14Δ	yGLuc+39c	yGLuc+43
A15Δ	yGLuc+42c	yGLuc+46
E16Δ	yGLuc+45c	yGLuc+49
A17Δ	yGLuc+48c	yGLuc+52
K18Δ	yGLuc+51c	yGLuc+55
P19Δ	yGLuc+54c	yGLuc+58
T20Δ	yGLuc+57c	yGLuc+61
T20-N23Δ	yGLuc+57c	yGLuc+70
E21-N23Δ	yGLuc+60c	yGLuc+70
E24-F26Δ	yGLuc+69c	yGLuc+79
E21Δ	yGLuc+60c	yGLuc+64
N22N23Δ	yGLuc+63c	yGLuc+70
N23Δ	yGLuc+66c	yGLuc+70
E24Δ	yGLuc+69c	yGLuc+73
D25Δ	yGLuc+72c	yGLuc+76
F26Δ	yGLuc+75c	yGLuc+79
N27Δ	yGLuc+78c	yGLuc+82
I28-A30Δ	yGLuc+81c	yGLuc+91
V31-S33Δ	yGLuc+90c	yGLuc+100

V31-A36Δ	yGLuc+90c	yGLuc+109
T37-D39Δ	yGLuc+108c	yGLuc+118
16EF	Fc-yGLuc+45c	yGLuc+49
16EL	Lc-yGLuc+45c	yGLuc+49
16EI	Ic-yGLuc+45c	yGLuc+49
16EM	Mc-yGLuc+45c	yGLuc+49
16EV	Vc-yGLuc+45c	yGLuc+49
16ES	Sc-yGLuc+45c	yGLuc+49
16EP	Pc-yGLuc+45c	yGLuc+49
16ET	Tc-yGLuc+45c	yGLuc+49
16EA	Ac-yGLuc+45c	yGLuc+49
16EY	Yc-yGLuc+45c	yGLuc+49
16EH	Hc-yGLuc+45c	yGLuc+49
16EQ	Qc-yGLuc+45c	yGLuc+49
16EN	Nc-yGLuc+45c	yGLuc+49
16EK	Kc-yGLuc+45c	yGLuc+49
16ED	Dc-yGLuc+45c	yGLuc+49
16EC	Cc-yGLuc+45c	yGLuc+49
16EW	Wc-yGLuc+45c	yGLuc+49
16ER	Rc-yGLuc+45c	yGLuc+49
16EG	Gc-yGLuc+45c	yGLuc+49
G2V3Δ4KF	MFc-TDH3-1c	yGLuc+13
G2V3Δ4KL	MLc-TDH3-1c	yGLuc+13
G2V3Δ4KI	MIc-TDH3-1c	yGLuc+13
G2V3Δ4KM	MMc-TDH3-1c	yGLuc+13
G2V3Δ4KV	MVc-TDH3-1c	yGLuc+13
G2V3Δ4KS	MSc-TDH3-1c	yGLuc+13
G2V3Δ4KP	MPc-TDH3-1c	yGLuc+13
G2V3Δ4KT	MTc-TDH3-1c	yGLuc+13
G2V3Δ4KA	MAc-TDH3-1c	yGLuc+13
G2V3Δ4KY	MYc-TDH3-1c	yGLuc+13
G2V3Δ4KH	MHc-TDH3-1c	yGLuc+13
G2V3Δ4KQ	MQc-TDH3-1c	yGLuc+13
G2V3Δ4KN	MNc-TDH3-1c	yGLuc+13
G2V3Δ4KD	MDc-TDH3-1c	yGLuc+13

G2V3ΔKE	MEc-TDH3-1c	yGLuc+13
G2V3ΔKC	MCc-TDH3-1c	yGLuc+13
G2V3Δ4KW	MWc-TDH3-1c	yGLuc+13
G2V3ΔKR	MRc-TDH3-1c	yGLuc+13
G2V3Δ4KG	MGc-TDH3-1c	yGLuc+13
KL^{8}	TDH3-1c40	MKL(8)-hGLuc+37
RL^{8}	TDH3-1c40	MRL(8)-hGLuc+37
RM^{8}	TDH3-1c40	MRM(8)-hGLuc+37
RF^{8}	TDH3-1c40	MRF(8)-hGLuc+37
RA^{8}	TDH3-1c40	MRA(8)-hGLuc+37
RW^{8}	TDH3-1c40	MRW(8)-hGLuc+37
RV^{8}	TDH3-1c40	MRV(8)-hGLuc+37
RY^{8}	TDH3-1c40	MRY(8)-hGLuc+37
RI^{8}	TDH3-1c40	MRI(8)-hGLuc+37
RS^{8}	TDH3-1c40	MRS(8)-hGLuc+37
RT^{8}	TDH3-1c40	MRT(8)-hGLuc+37
RQ^{8}	TDH3-1c40	MRQ(8)-hGLuc+37
RC^{8a}	TDH3-1c40	MRC(8)-hGLuc+37
L^{7}	TDH3-1c40	MKL(7)-hGLuc+46
L^{8}	TDH3-1c40	MKL(8)-hGLuc+46
L^{9}	TDH3-1c40	MKL(9)-hGLuc+46
L^{10}	TDH3-1c40	MKL(10)-hGLuc+46
L^{11}	TDH3-1c40	MKL(11)-hGLuc+46
L^{12}	TDH3-1c40	MKL(12)-hGLuc+46
L^{13}	TDH3-1c40	MKL(13)-hGLuc+46
L^{14}	TDH3-1c40	MKL(14)-hGLuc+46
L^{15}	TDH3-1c40	MKL(15)-hGLuc+46
L^{16}	TDH3-1c40	MKL(16)-hGLuc+46
L^{17}	TDH3-1c40	MKL(17)-hGLuc+46
$L^{13}F$	TDH3-1c40	MKL(13)F-hGLuc+46
$L^{13}L$	TDH3-1c40	MKL(13)L-hGLuc+46
$L^{13}I$	TDH3-1c40	MKL(13)I-hGLuc+46
$L^{13}M$	TDH3-1c40	MKL(13)M-hGLuc+46
$L^{13}V$	TDH3-1c40	MKL(13)V-hGLuc+46
$L^{13}S$	TDH3-1c40	MKL(13)S-hGLuc+46

$L^{13}P$	TDH3-1c40	MKL(13)P-hGLuc+46
$L^{13}T$	TDH3-1c40	MKL(13)T-hGLuc+46
$L^{13}A$	TDH3-1c40	MKL(13)A-hGLuc+46
$L^{13}Y$	TDH3-1c40	MKL(13)Y-hGLuc+46
$L^{13}H$	TDH3-1c40	MKL(13)H-hGLuc+46
$L^{13}Q$	TDH3-1c40	MKL(13)Q-hGLuc+46
$L^{13}N$	TDH3-1c40	MKL(13)N-hGLuc+46
$L^{13}K$	TDH3-1c40	MKL(13)K-hGLuc+46
$L^{13}D$	TDH3-1c40	MKL(13)D-hGLuc+46
$L^{13}C$	TDH3-1c40	MKL(13)C-hGLuc+46
$L^{13}W$	TDH3-1c40	MKL(13)W-hGLuc+46
$L^{13}R$	TDH3-1c40	MKL(13)R-hGLuc+46
$L^{13}G$	TDH3-1c40	MKL(13)G-hGLuc+46
$L^{13}F$	TDH3-1c40	MKL(13)F-hGLuc+46
$L^{13}L$	TDH3-1c40	MKL(13)L-hGLuc+46
$L^{13}I$	TDH3-1c40	MKL(13)I-hGLuc+46
$L^{13}M$	TDH3-1c40	MKL(13)M-hGLuc+46
$L^{13}V$	TDH3-1c40	MKL(13)V-hGLuc+46
$L^{13}S$	TDH3-1c40	MKL(13)S-hGLuc+46
$L^{13}P$	TDH3-1c40	MKL(13)P-hGLuc+46
$L^{13}T$	TDH3-1c40	MKL(13)T-hGLuc+46
$L^{13}A$	TDH3-1c40	MKL(13)A-hGLuc+46
$L^{13}Y$	TDH3-1c40	MKL(13)Y-hGLuc+46
$L^{13}H$	TDH3-1c40	MKL(13)H-hGLuc+46
$L^{13}Q$	TDH3-1c40	MKL(13)Q-hGLuc+46
$L^{13}N$	TDH3-1c40	MKL(13)N-hGLuc+46
$L^{13}K$	TDH3-1c40	MKL(13)K-hGLuc+46
$L^{13}D$	TDH3-1c40	MKL(13)D-hGLuc+46
$L^{13}C$	TDH3-1c40	MKL(13)C-hGLuc+46
$L^{13}W$	TDH3-1c40	MKL(13)W-hGLuc+46
$L^{13}R$	TDH3-1c40	MKL(13)R-hGLuc+46
$L^{13}G$	TDH3-1c40	MKL(13)G-hGLuc+46
I^{7a}	TDH3-1c40	MKI(7)-hGLuc+46
I^{8a}	TDH3-1c40	MKI(8)-hGLuc+46
I^{9a}	TDH3-1c40	MKI(9)-hGLuc+46

I^{10}	TDH3-1c40	MKI(10)-hGLuc+46
I^{11}	TDH3-1c40	MKI(11)-hGLuc+46
I^{12}	TDH3-1c40	MKI(12)-hGLuc+46
I^{13}	TDH3-1c40	MKI(13)-hGLuc+46
I^{14}	TDH3-1c40	MKI(14)-hGLuc+46
I^{15}	TDH3-1c40	MKI(15)-hGLuc+46
I^{16}	TDH3-1c40	MKI(16)-hGLuc+46
I^{17}	TDH3-1c40	MKI(17)-hGLuc+46
F^{7a}	TDH3-1c40	MKF(7)-hGLuc+46
F^{8}	TDH3-1c40	MKF(8)-hGLuc+46
F^{9}	TDH3-1c40	MKF(9)-hGLuc+46
F^{10}	TDH3-1c40	MKF(10)-hGLuc+46
F^{11}	TDH3-1c40	MKF(11)-hGLuc+46
F^{13}	TDH3-1c40	MKF(13)-hGLuc+46
F^{15}	TDH3-1c40	MKF(15)-hGLuc+46
F^{17}	TDH3-1c40	MKF(17)-hGLuc+46
M^{7a}	TDH3-1c40	MKM(7)-hGLuc+46
M^{8a}	TDH3-1c40	MKM(8)-hGLuc+46
M^{9a}	TDH3-1c40	MKM(9)-hGLuc+46
M^{10}	TDH3-1c40	MKM(10)-hGLuc+46
M^{11}	TDH3-1c40	MKM(11)-hGLuc+46
M^{12}	TDH3-1c40	MKM(12)-hGLuc+46
M^{13}	TDH3-1c40	MKM(13)-hGLuc+46
M^{14}	TDH3-1c40	MKM(14)-hGLuc+46
M^{15}	TDH3-1c40	MKM(15)-hGLuc+46
M^{16}	TDH3-1c40	MKM(16)-hGLuc+46
M^{17}	TDH3-1c40	MKM(17)-hGLuc+46
AoTAA	TDH3-1c40	TAA+(1-54)-hGLuc+49
SfGLU1	TDH3-1c40	SfGLU1+(1-54)-hGLuc+49
BlAmyL	AmyLN5C+(1-27)c-TDH3-1c	AmyLN5C+(28-69)-hGLuc+49
KmPGU1	TDH3-1c	KmPGU1+(1-54)-hGLuc+49
hIL6	hIL6+(1-33)c-TDH3-1c	hIL6+(34-69)-hGLuc+49
hEPO	hEPO+(1-21)c-TDH3-1c	hEPO+(22-60)-yGLuc+49
hLIF	hLIF+(1-30)c-TDH3-1c	hLIF+(31-78)-yGLuc+49
hAZGP1	hZA2G+(1-18)c-TDH3-1c [b]	hZA2G+(19-51)-yGLuc+49 [b]

[a]These constructs showed the same level of null value, thus data were not shown in the result. [b]hZA2G is an alternative name for hAZGP1.
